# Ascending aortic extension to increase aortopulmonary space after comprehensive stage II palliation

**DOI:** 10.1093/icvts/ivab345

**Published:** 2021-12-09

**Authors:** Gianluca Brancaccio, Matteo Trezzi, Aurelio Secinaro, Roberta Iacobelli, Gianluigi Perri, Sergio Filippelli, Veronica Bordonaro, Lorenzo Galletti

**Affiliations:** 1 Department of Pediatric Cardiology and Cardiac Suegery, Ospedale Pediatrico bambino Gesù IRCCS, Rome, Italy; 2 Department of Imaging, Cardio-Thoracic Imaging Unit, Ospedale Pediatrico Bambino Gesù IRCCS, Rome, Italy

**Keywords:** Hybrid palliation, Hypoplastic Left heart syndrome, Left pulmonary artery

## Abstract

Aortic reconstruction at the time of the comprehensive stage II (CSII) procedure can be complicated by compression within the aortopulmonary space resulting in airway or pulmonary artery narrowing. We describe our experience with 2 patients with hypoplastic left heart syndrome and pulmonary artery stenosis after the CSII procedure. Both patients underwent an aortic extension with a Hemashield interposition graft to open up the aortopulmonary space. The patients were discharged from the hospital. In all cases the aortopulmonary space was enlarged, and the pulmonary arteries and airway were free from compression. Aortic extension is an option to be considered in children with pulmonary artery compression who previously had a CSII procedure.

## INTRODUCTION

Aortic reconstruction after a Norwood operation is crucial for the long-term outcome of patients with hypoplastic left heart syndrome (HLHS). Hybrid palliation has postponed aortic reconstruction from the neonatal period to ∼6 months of age [1]. However, pulmonary artery banding can cause the pulmonary artery (PA) trunk to dilate, making aortic reconstruction wider and causing a reduction of the aortopulmonary space (APS) that can potentially narrow the airways or the PAs [2, 3].

We report our experience with 2 children who underwent aortic extension to open up the APS because of left PA or left bronchial compression.

## DESCRIPTION

### Case 1

**Figure 1:**
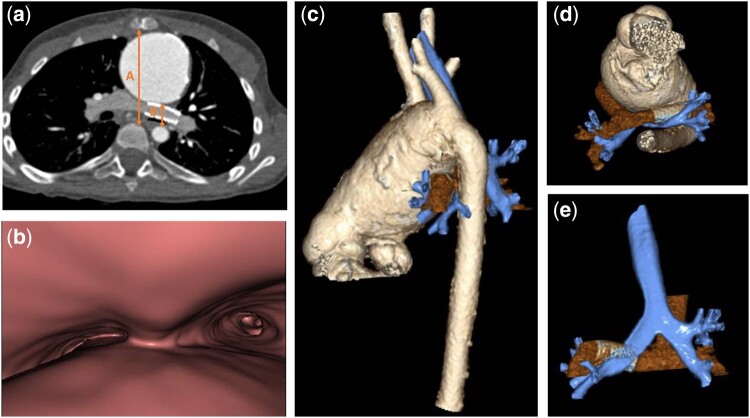
Preoperative computed tomography. Axial contrast-enhanced computed tomography image (**a**) with virtual bronchoscopy (**b**) and 3-dimensional volume rendering reconstructions (**c**–**e**) show a severely enlarged ascending aorta with posterior displacement of the stented left pulmonary artery and marked left main bronchus compression. The interaortic space from the posterior margin of the ascending aorta to the anterior margin of the descending aorta (distance *B* in **a**) is critically narrowed, with a low value of the interaortic ratio (*B*/*A*).

**Figure 2:**
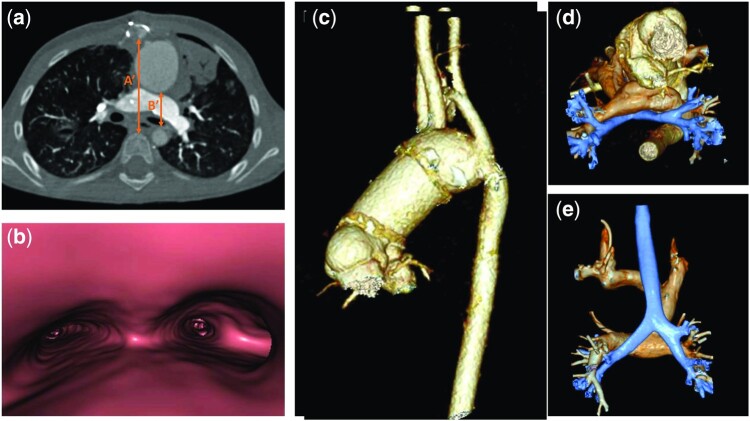
Postoperative computed tomography. The postoperative computed tomography scan (**a**) shows the appearance of the ascending aorta after surgical elongation with interposition of the Hemashield graft (**c-e**) and the resulting enlargement of the aortopulmonary space with relief of the main bronchus obstruction, as evident in virtual bronchoscopy view (**b**). This surgical approach allowed a considerable increase of the interaortic ratio (*B*′/*A*′) compared to the preoperative computed tomography scan.

Shortly after birth, 1 patient (HLHS syndrome with mitral atresia and aortic stenosis) underwent neonatal hybrid palliation with ductal stenting and bilateral pulmonary artery banding. At 8 months of age, the patient underwent comprehensive stage II (CSII). The postoperative course was complicated by elevated pressure in the cavopulmonary system (22 mmHg), and cardiac catheterization showed left PA narrowing caused by a dilated neoaorta. Therefore, on the second postoperative day the patient underwent left PA stenting that reduced the cavopulmonary pressure with good resolution of the left PA stenosis. Following an extended period during which the patient did not have any follow-up appointments, she was seen in our outpatient clinic at the age of 5; echocardiography showed severe tricuspid valve insufficiency. Therefore, before the Fontan completion, tricuspid valve repair as an interstage procedure was deemed necessary. A preoperative computed tomography (CT) scan showed posterior displacement of the stented left pulmonary artery. Compression by the enlarged ascending aorta was responsible for obstructing the left main bronchus. Moreover, a severe reduction of the interaortic (IA) distance [4, 5], measured from the posterior margin of the ascending aorta to the anterior margin of descending aorta, with a low IA ratio (0.24; IA length from the sternum to the spine) was found (Fig.1a–e). Magnetic resonance imaging evaluation revealed an asymmetrical flow distribution (right pulmonary artery/left pulmonary artery: 67%/37%). The patient underwent redo sternotomy. Extensive mobilization of the aorta and aortic arch and the pulmonary arteries was necessary. The aorta was transected, and the pulmonary arteries were augmented. The stent was discontinued, utilizing a pulmonary homograft patch. The aorta was reconstructed and elongated with interposition of a 28-mm Hemashield graft to enlarge the APS. A brief period of antegrade cerebral perfusion at 24°C was necessary for the distal arch anastomosis. Concomitant tricuspid valve repair with partial annuloplasty was performed. The patient experienced a difficult postoperative course with cyanosis. After a brief period of extracorporeal membrane oxygenation, the patient required an axillary arterio-venous fistula to increase the arterial saturation. A postoperative CT scan demonstrated a wide open left main stem bronchus, with a considerable increase in the IA ratio (0.36) (Fig. 2a–e). The patient was discharged to home and is waiting for the Fontan completion.

### Case 2

Following a neonatal hybrid palliation at 7 months of age, patient # 2 underwent CSII and at 3 years of age, the fenestrated Fontan completion. The night after the Fontan, he experienced values up to 21 mmHg in the Fontan circuit and underwent emergency left pulmonary artery (LPA) stenting for LPA stenosis. The day after, he developed acute LPA thrombosis and required a Fontan take-down, with stent removal and left PA augmentation. At 7 years of age, a CT scan revealed a tapered aspect of the left PA with calibre reduction towards the hilum, where it runs between the aortic arch and the left main bronchus.

Cardiac catheterization revealed a pressure of 12 mmHg in the cavopulmonary system. The patient was referred for a redo Fontan operation with LPA augmentation and aortic elongation in order to augment the APS. After clamping and dividing the aorta, extensive mobilization of the aortic arch and ascending aorta was required. The aorta was elongated with the interposition of a 28-mm Hemashield graft. A brief period of anterograde cerebral perfusion at 24°C was necessary for distal arch anastomosis. The operation was completed with the interposition of a 20-mm Gore-Tex (W. L. Gore Inc., Newark, DE, USA) graft from the inferior vena cava and the PA, and a 4-mm fenestration was done. The postoperative course was uneventful with a Fontan pressure of 14 mmHg.

## COMMENT

Different authors have reported that the “Achilles heel” of Giessen HP is the high interventional requirement for LPA stenosis due to the narrowing of the APS after aortic reconstruction [2, 3]. PA compression is especially problematic in children with stoke volume physiology where an increase in pulmonary vascular resistance may impair systemic venous flow and reduce cardiac output [2]. Following the CSII procedure, PA-related interventions vary from 50% to 86% compared with 16–31% after the Norwood procedure [2, 3]. Furthermore, association of the bidirectional cavopulmonary connection at the time of the Damus-Kaye-Stansel procedure leads to PAs perfused by a passive low-pressure venous flow. Therefore LPA is more susceptible to external compression by the pulsatile enlarged neoaorta. Consequently, we recently abandoned the routine practice of hybrid palliation in the treatment of HLHS, which we currently reserve only for low-weight patients, patients with several comorbidities or patients with borderline LV as a ‘bridge to decision’ towards a biventricular or univentricular pathway.

The usual approach to treat aortic compression of structures in the APS has been aortopexy [6]. However, anterior suspension of the aorta to the chest wall may not be successful after the Norwood procedure and may complicate later sternal re-entry. Another possible approach is percutaneous LPA stenting [7], but it may cause tracheobronchial compression with the possibility of erosion of the PA wall in contiguity with aortic reconstruction. Ascending aortic elongation procedures have been previously reported to relieve LPA or airway stenosis for APS narrowing, especially after interrupted aortic arch and/or truncus arteriosus repair [8, 9]. Furthermore, large aortic reconstructions allow the use of adult-sized grafts without the need for further replacements. In our patients, cerebral perfusion through a shunt in the innominate artery was utilized to avoid aortic cross-clamping and to allow a large anastomosis free of tension.

Our current strategies in patients palliated with the hybrid approach is to elongate the aorta in all patients who preoperatively showed an IA <0.25.

In conclusion, aortic extension is an option to be considered in children with left pulmonary stenosis undergoing CSII.

## Reviewer information

Interactive CardioVascular and Thoracic Surgery thanks Tim Attmann and the other, anonymous reviewer(s) for their contribution to the peer review process of this article.

## Funding

This study was performed without external financial support.

**Conflict of interest:** None declared.
